# Genome-Wide Identification of the SWEET Gene Family and Functional Analysis of *BraSWEET10* in Winter *B. rapa* (*Brassica rapa* L.) Under Low-Temperature Stress

**DOI:** 10.3390/ijms26062398

**Published:** 2025-03-07

**Authors:** Jinli Yue, Shunjie Yuan, Lijun Liu, Zaoxia Niu, Li Ma, Yuanyuan Pu, Junyan Wu, Yan Fang, Wancang Sun

**Affiliations:** 1 State Key Laboratory of Aridland Crop Science/College of Agronomy, Gansu Agricultural University, Lanzhou 730070, Chinaliulj@gsau.edu.cn (L.L.); puyy@gsau.edu.cn (Y.P.);; 2 Gansu Vocational College of Agriculture, Lanzhou 730020, China; 3 Lanzhou Institute for Food and Drug Control, Lanzhou 730070, China; 4 Institute of Crop, Gansu Academy of Agricultural Sciences, Lanzhou 730070, China

**Keywords:** *Brassica rapa* L., SWEET gene family, *SWEET 10*, low-temperature stress

## Abstract

Sugars will eventually be exported transporter (SWEET), a class of glucose transport proteins, is crucial in plants for glucose transport by redistribution of sugars and regulates growth, development, and stress tolerance. Although the SWEET family has been studied in many plants, little is known about its function in winter *B. rapa* (*Brassica rapa* L.). Bioinformatics approaches were adopted to identify the SWEET gene (*BraSWEETs*) family in *B. rapa* to investigate its role during overwintering. From the whole-genome data, 31 *BraSWEET* genes were identified. Gene expansion was realized by tandem and fragment duplication, and the 31 genes were classified into four branches by phylogenetic analysis. As indicated by exon–intron structure, cis-acting elements, MEME (Multiple EM for Motif Elicitation) motifs, and protein structure, *BraSWEETs* were evolutionarily conserved. According to the heat map, 23 *BraSWEET* genes were differentially expressed during overwintering, revealing their potential functions in response to low-temperature stress and involvement in the overwintering memory-formation mechanism. *BraSWEET10* is mainly associated with plant reproductive growth and may be crucial in the formation of overwintering memory in *B. rapa*. The *BraSWEET10* gene was cloned into *B. rapa* (Longyou-7, L7). The BraSWEET10 protein contained seven transmembrane structural domains. Real-time fluorescence quantitative PCR (qRT-PCR) showed that the *BraSWEET10* gene responded to low-temperature stress. BraSWEET10 was localized to the cell membrane. The root length of overexpressing transgenic *A. thaliana* was significantly higher than that of wild-type (WT) *A. thaliana* under low temperatures. Our findings suggest that this gene may be important for the adaptation of winter *B. rapa* to low-temperature stress. Overall, the findings are expected to contribute to understanding the evolutionary links of the BraSWEET family and lay the foundation for future studies on the functional characteristics of *BraSWEET* genes.

## 1. Introduction

Sugar transport and homeostasis are crucial for regulating plant growth, development, and responses to biotic and abiotic stress. The content of soluble sugars, including sucrose, glucose, fructose, and galactose, varies in plants during freezing resistance, and it is an important physiological indicator directing freezing resistance in plants [1].

In plants, sugars are produced by the photosynthetic tissue (source) and then transported to non-autotrophic tissue (reservoir). This process involves sugar transport across cell organelles, transport across different cells, and long-distance transport through the phloem. During transport, sugars need to traverse through organelle membranes and cell membranes several times [2], and sugar transporters are pivotal in these processes. This implies that sugar transporters are key factors regulating the redistribution of soluble sugars and can respond to various stresses [1]. To date, several membrane-located sugar transporters have been identified in plants, including the sucrose transporters (SUTs) family [3], monosaccharide transporters (MSTs) [4,5], and the sugars will eventually be exported transporter (SWEET) family [6].

SWEET proteins are a newly identified class of sugar efflux transporters. As pH-independent bidirectional transporters of sugars, SWEET proteins can facilitate the diffusion of various soluble sugars to the apoplast on cell efflux by crossing the cell membrane following the concentration gradient, and they function as low-affinity glucose transport proteins to regulate glucose uptake across the cell membrane [7,8]. In eukaryotes, such as humans, nematodes, ascidians, and plants, the SWEET transporter protein comprises seven trans-membrane α-helices (trans-membrane helix (TMH)). At the N terminal and C terminal of the SWEET protein, there is an MtN3/saliva structural domain, which comprises two conservative 3-TMH, resultant of tandem duplication. The remaining TMH serves as a link in the middle, thus forming a “3-1-3” structure [6].

The number of the SWEET gene family members varies in different species and has not been implicated in the complexity of species evolution [8]. For example, the Chlamydomonas SWEET gene family comprises three genes, while the higher creature (human) and mice contain only one gene separately [7]. In contrast, vascular plants contain multiple *SWEET* genes. For instance, the SWEET family has 17, 21, and 15 members in *A. thaliana*, rice, and alfalfa, respectively [9]. *SWEETs* exhibit functional diversity in plants. According to relevant studies, genes in this family are involved in many biological processes, such as sugar transport, ion transport, host–pathogen interaction, plant development, senescence, and stress resistance.

In recent years, studies on the relationship between *SWEETs* and abiotic stress resistance in plants have mainly focused on *A. thaliana*. Many *SWEET* genes in different plants are induced by abiotic stress at the transcriptional level, indicating their possible association with plant response to stress. *AtSWEET15* is related to plant senescence and is involved in mediating response to abiotic stress. The expression of *AtSWEET15* is induced by cold, high salt, and drought via the ABA-dependent pathway, and is upregulated under cold stress. *AtSWEET15* overexpression lines are more sensitive to salt stress, and mutants exhibit higher root cell viability under salt stress compared with the control [10]. *AtSWEET17*, a tonoplast-located fructose transporter, can mediate fructose transport in vacuoles. The *AtSWEET17* mutant accumulates more fructose under nitrogen deficiency and cold stress, suggesting that *AtSWEET17* is involved in plant stress resistance [11,12]. *AtSWEET16*, a homolog of *AtSWEET17,* is downregulated in leaves under cold stress, osmotic stress, and low-nitrogen stress. Under cold stress, the overexpression lines of *AtSWEET16* show increased fructose concentration in leaves, enhanced root growth, increased plant cold tolerance, and improved nitrogen use efficiency [11]. *AtSWEET11* and *AtSWEET12* participate in cold stress tolerance by regulating the number and pore diameter of xylem vessels [13]. During cold acclimation, the expressions of *CsSWEET2, 3,* and *6* are notably inhibited, while those of *CsSWEET1* and *CsSWEET17* increase sharply. Under high-salt and cold stress, the induced expression of *SWEETs* in other plants, such as barley and tomato, also supports the involvement of *SWEET* genes in regulating abiotic stress [8].

In northern China, winter *B. rapa* is the only overwintering oil crop [14]. In the cold winter, winter *B. rapa* can resist the cold environment by preserving its roots and the shoot apical meristem [15]. A systematic study on the SWEET gene family of winter *B. rapa* is lacking. Owing to its high homology, reports on the SWEET gene family have focused on its role in reproductive development and the clubroot of Brassica crops. Several *SWEET* genes belonging to branches I and III show remarkable upregulation during *Plasmodiophora Brassicae*-induced formation [16]. *BraSWEET9/BcNS* is crucial in development, particularly that of the floral nectary [17]. Nonetheless, a relevant study on the function of the *SWEET* genes in stress resistance of *B. rapa* is lacking. In this study, Longyou-7 (L7) (cold tolerances) was chosen as the experimental object, and we focused on the phylogeny, gene structure, chromosome distribution, and cis-acting regulatory elements of the SWEET family. Simultaneously, *BraSWEET* gene expression during overwintering was analyzed. This study aimed to explore the important function of the BraSWEET gene family in the excellent freezing resistance from the perspective of gene evolution and structure in *B. rapa*. It provides valuable information for studies on improved freezing resistance in *B. rapa*.

## 2. Results

### 2.1. Identification and Structural Analysis of BraSWEET Genes Family

Thirty-one *SWEET* genes were identified in the genome of *B. rapa* (Table 1), 1.82 times that of the AtSWEET gene family. Among the 31 *BraSWEET* genes, Brapa05T003594.1/BraSWEET8.1 belongs to the semi-SWEET group containing only one MtN3 domain, and all other proteins contain two MtN3 domains [18]. Except for *Brapa05T003594.1/BraSWEET8.1*, the CDS (Coding DNA Sequence) lengths of the genes ranged from 686 (*BraSWEET16.1*) to 1274 bp (*BraSWEET11.2*); the encoded protein lengths varied from 205 to 298 amino acid residues; molecular weights (MW) were between 22.813 kDa (BraSWEET4.1) and 33.384 kDa (BraSWEET15.2); and the predicted isoelectric (PI) values varied from 7.66 (BraSWEET4.1) to 9.54 (BraSWEET6.1). A comparison of the homology of the 17 *AtSWEET* genes and 31 *BraSWEET* genes is shown in Table 1.

**Table 1:** Characteristics of *SWEET* genes in *B. rapa* and information relevant to *A. thaliana*.

Gene Name	Gene ID	Chromosome Number	Transmembrane Domains	CDS (bp)	Protein Length (aa)	MW (kD)	PI	Number of Domains
*BraSWEET1.1*	*Brapa06T001655.1*	A06	7	948	246	27.155	9.3	2
*BraSWEET1.2*	*Brapa08T002789.1*	A08	7	1006	250	27.66	9.29	2
*BraSWEET2*	*Brapa05T002998.1*	A05	7	1210	236	26.644	8.95	2
*BraSWEET2.1*	*Brapa01T003748.1*	A01	7	969	236	26.3626	8.93	2
*BraSWEET3*	*Brapa02T001534.1*	A02	6	799	242	27.584	8.52	2
*BraSWEET4.1*	*Brapa02T003964.1*	A02	6	688	205	22.813	7.66	2
*BraSWEET4.2*	*Brapa06T003316.1*	A06	8	1183	297	32.983	9.22	2
*BraSWEET5*	*Brapa09T005858.1*	A09	7	723	240	27.244	9.04	2
*BraSWEET5.1*	*Brapa02T004551.1*	A02	7	723	240	26.971	8.15	2
*BraSWEET5.2*	*Brapa02T004552.1*	A02	7	723	240	26.823	8.14	2
*BraSWEET6.1*	*Brapa09T003889.1*	A09	7	741	246	27.215	9.54	2
*BraSWEET6.2*	*Brapa03T002763.1*	A03	6	789	262	28.707	9.25	2
*BraSWEET8*	*Brapa04T001417.1*	A04	6	717	238	26.794	8.97	2
*BraSWEET8.1*	*Brapa05T003594.1*	A05	1	189	62	7.012	4.66	1
*BraSWEET8.2*	*Brapa05T003592.1*	A05	6	924	242	27.207	8.42	2
*BraSWEET9*	*Brapa03T002066.1*	A03	7	813	270	30.125	9.18	2
*BraSWEET10*	*Brapa03T001544.1*	A03	7	952	289	33.042	9.29	2
*BraSWEET11.1*	*Brapa01T002415.1*	A01	7	1197	290	32.344	9.04	2
*BraSWEET11.2*	*Brapa06T001802.1*	A06	7	1274	289	32.067	9.25	2
*BraSWEET12.1*	*Brapa06T002739.1*	A06	7	1318	288	31.782	9.07	2
*BraSWEET12.2*	*Brapa09T005963.1*	A09	7	1146	277	30.498	9.13	2
*BraSWEET14*	*Brapa03T005270.1*	A03	7	1048	273	30.29	9.27	2
*BraSWEET14.1*	*Brapa08T002066.1*	A08	7	930	272	29.917	9.18	2
*BraSWEET14.2*	*Brapa01T001548.1*	A01	7	1079	273	30.1	9.2	2
*BraSWEET15.1*	*Brapa03T000563.1*	A03	7	1057	292	32.848	8.16	2
*BraSWEET15.2*	*Brapa10T000811.1*	A10	7	1155	298	33.384	8.44	2
*BraSWEET15.3*	*Brapa02T000470.1*	A02	7	1129	297	33.156	8.26	2
*BraSWEET16.1*	*Brapa03T003796.1*	A03	7	686	231	25.68	9.06	2
*BraSWEET16.2*	*Brapa01T003622.1*	A01	7	696	231	25.755	8.69	2
*BraSWEET17.1*	*Brapa08T001162.1*	A08	7	1001	240	26.561	9.24	2
*BraSWEET17.2*	*Brapa03T004706.1*	A03	7	1182	240	26.455	8.76	2

**Figure 1:**
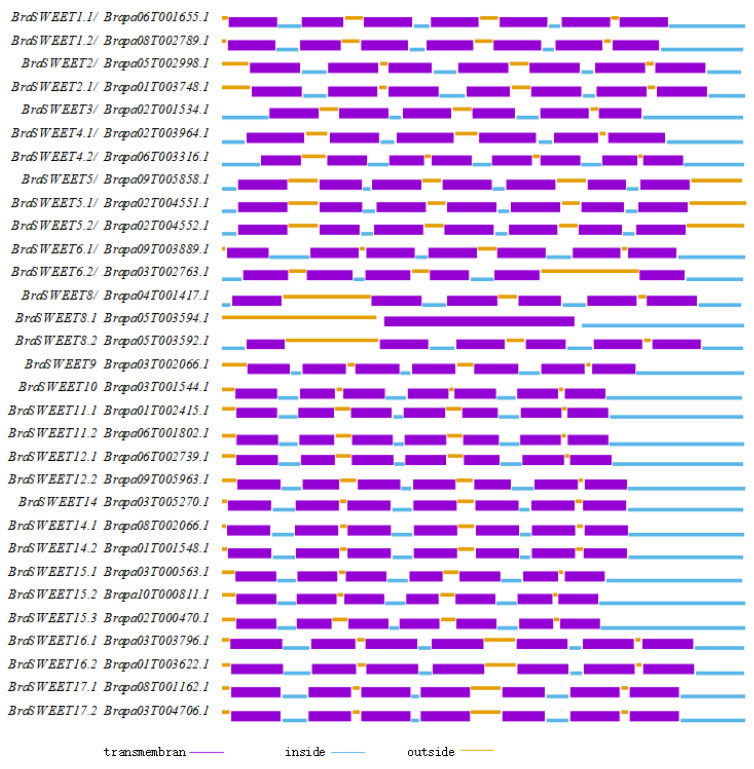
The transmembrane domain of BraSWEET proteins. The blue lines signify the intracellular region. The thick purple line denotes the transmembrane region. Yellow lines indicate the extracellular region.

The trans-membrane regions of 31 BraSWEET proteins were predicted using the TMHMM2.0 (http://www.cbs.dtu.dk/services/TMHMM/ (accessed on 5 March 2025)) software. Twenty-four members of the BraSWEET family had seven typical trans-membrane helixes (Figure 1), similar to the characteristics of SWEET family members in other species containing seven trans-membrane helixes and two MtN3 domains. Five members had six trans-membrane helixes; BraSWEET8.1/Brapa05T003594.1 contained only one trans-membrane domain; and BraSWEET4.2/Brapa06T003316.1 included eight trans-membrane domains. Accordingly, BraSWEET family proteins may be localized on the membrane as receptors.

**Figure 2:**
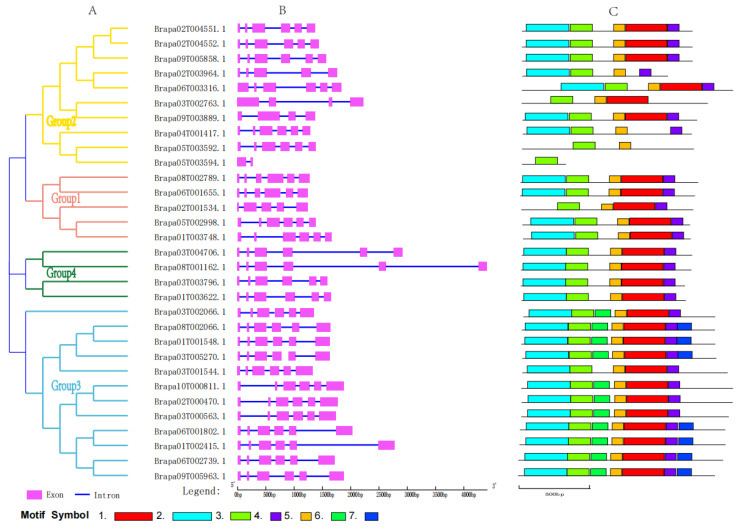
Gene structure and motifs of the *BraSWEET* genes. (**A**) The phylogenetic tree of BraSWEET proteins. (**B**) The exon–intron structure of 31 *BraSWEET* genes. Exons and introns are represented by rose boxes and blue lines, respectively. (**C**) The motif composition of BraSWEET proteins. The seven motifs are represented by differently colored rectangles.

Intron–exon mapping analysis showed a highly conserved number and distribution of introns and exons of *SWEET* genes in *B. rapa* (Figure 2B). Six exons are contained in 27 members of the BraSWEET family, five in the *BraSWEET3/Brapa02T001534.1*, four in two members (*BraSWEET6.1/Brapa09T003889.1* and *BraSWEET6.2/Brapa03T002763.1*), and two in the shortest, *BraSWEET8.1/Brapa05T003594.1.*

Using the MEME program, conserved motifs of 31 *BraSWEETs* were predicted. Based on the *BraSWEETs’* sequence characteristics, seven motifs, designated motifs 1–7, were identified and exhibited (Figure 2C and Figure S1). Thirty-one members contained motif 3. Except for *BraSWEET8.1/Brapa05T003594.1*, the remaining genes contained motif 5. *BraSWEET8/Brapa04T001417.1* and *BraSWEET4.1/Brapa02T003964.1* contained motifs 2, 3, 4, and 6. *BraSWEET3/Brapa02T001534.1* contained motifs 1, 3, 4, and 5. In combination with the clade, Group 1, Group 3, and Group 4 had conserved motifs. For example, motifs 6 and 7 were only found in Group 3. The number and distribution of motifs in Group 2 differed. *BraSWEET8.1/Brapa05T003594.1*, *BraSWEET8.2/Brapa05T003592.1*, and *BraSWEET6.2/Brapa03T002763.1* contained fewer motif types, and other homolog proteins had similar motif composition and distribution. Similar motif compositions in the same clade are indicative of the functional similarity of the members of each clade.

### 2.2. Phylogenetic Analysis of BraSWEET Proteins

**Figure 3:**
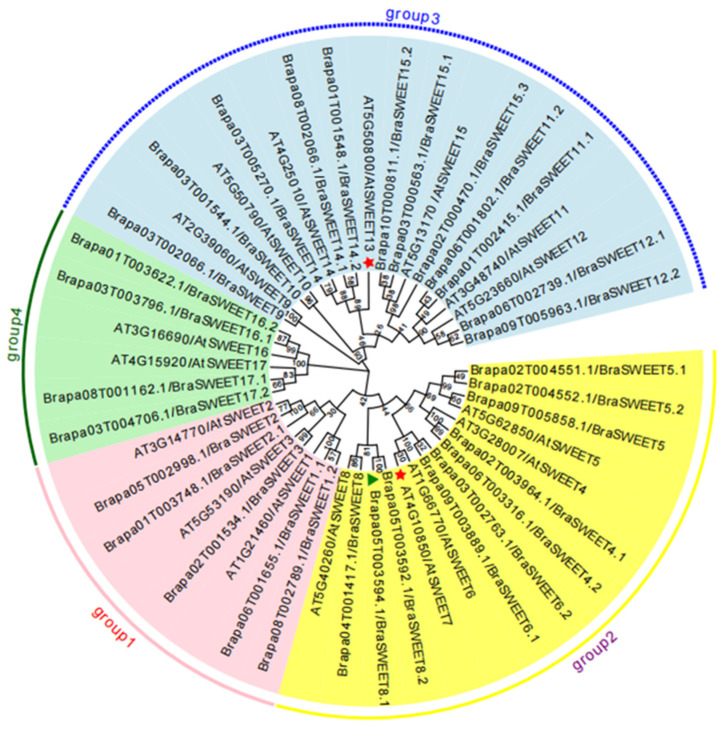
Phylogenetic tree of SWEET proteins in *Brassica rapa* L. (L7) and *A. thaliana*. The numbers on the branches indicate the bootstrap percentage values calculated from 1000 replicates. The genes in the pink, yellow, blue, and green clades are clubbed in Group1, Group2, Group3, and Group4, respectively. The clades containing only *AtSWEET* genes are marked with a red star. The clade containing only one MtN3 motif is indicated using a green triangle.

*B. rapa* and *A. thaliana* belong to the Brassicae family. By aligning using the MEGA VII (v7.0.14) software, a phylogenetic tree was constructed for the 31 BraSWEET and 17 AtSWEET amino acid sequences (Figure 3). According to the phylogenetic tree, BraSWEET proteins were classified into four clades, namely Group 1, Group 2, Group 3, and Group 4, respectively. Each clade contained 5, 10, 12, and 4 members of the BraSWEET family, respectively. Notably, it contained no corresponding proteins in *B. rapa* which were evolutionarily close to AtSWEET7 and AtSWEET13.

### 2.3. Chromosomal Localization, Collinearity Analysis, and Tandem Replication of BraSWEETs

**Figure 4:**
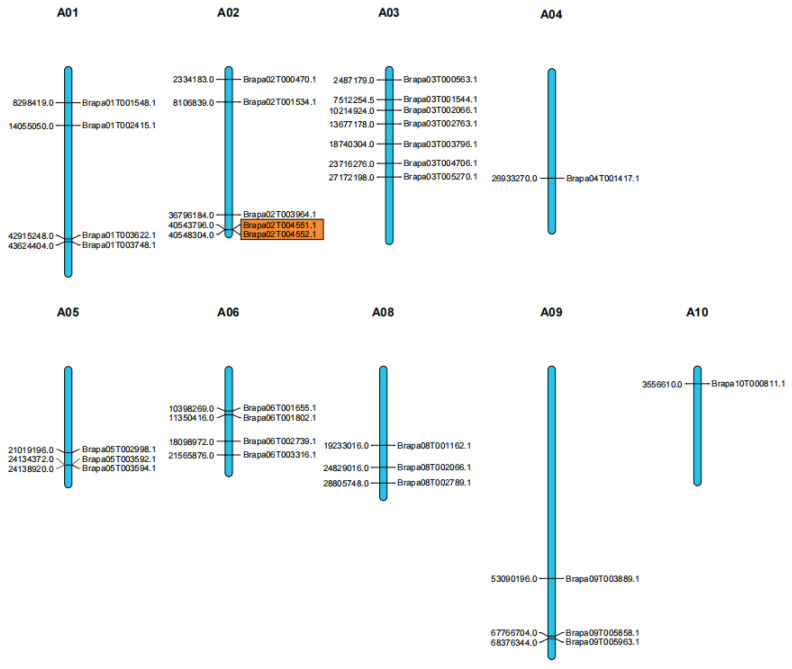
Chromosomal locations of *BraSWEET* genes. Black lines represent the gene position on the chromosome. Tandemly duplicated genes are indicated with orange boxes.

Thirty-one *BraSWEET* genes were distributed on 9 of 10 chromosomes (Figure 4). Seven genes were distributed on chromosome 3, the most among all chromosomes. There were four *BraSWEET* genes on chromosomes 1 and 6, five on chromosome 2, three on chromosomes 5, 8, and 9, and only one gene on chromosomes 4 and 10. There were no *BraSWEET* genes on chromosome 7. As shown by chromosomal localization analysis of SWEET family genes, the distribution of the *BraSWEET* genes on chromosomes was random and throughout the genome, rather than concentrated on a small number of chromosomes.

**Figure 5:**
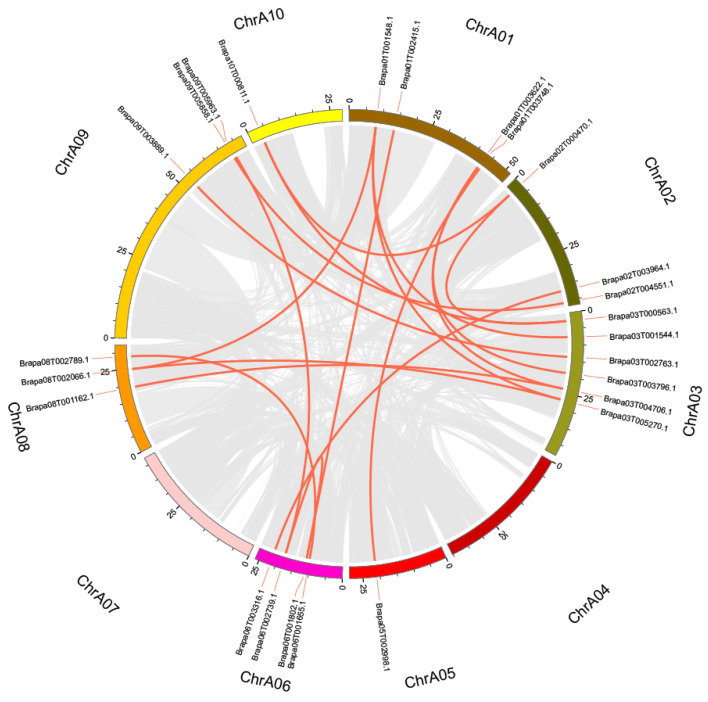
Synteny analysis for the SWEET family in *B. rapa* (L7). Gray lines indicate all synteny blocks in the genome of *B. rapa* (L7). Red lines indicate the duplication of *BraSWEET* gene pairs.

Gene duplication, including tandem and segmental duplication, is crucial for plant genome evolution. Only one pair of *BraSWEET* genes (*BraSWEET5.1/Brapa02T004551.1*, *BraSWEET5.2/Brapa02T004552.1*) was identified as tandem duplication and was located on chromosome 2 (Figure 4). There is a close genetic distance between *BraSWEET8.1/Brapa05T003594.1* and *BraSWEET8.2/Brapa05T003592.1*, but the length of *BraSWEET8.1* is merely 189 bp, which is less than 70% of the length of the *BraSWEET8.2* gene. Thus, it was not considered tandem duplication. A total of 18 fragment duplications were identified, all located on different chromosomes (Figure 5). Thus, gene replication is pivotal for the expansion of the BraSWEET gene family.

The rates of synonymous substitution (Ks) and non-synonymous substitution (Ka) were calculated, along with the Ka/Ks ratio of 19 pairs of duplicated genes to further understand the duplications of *BraSWEET* genes (Table 2). The Ka/Ks ratio is an important indicator of the selection pressure of the evaluated genes. Values of Ka/Ks = 1 indicate neutral selection, Ka/Ks < 1 denotes the purifying selection, and Ka/Ks > 1 signifies positive selection for acceleration of evolution [19]. The Ka/Ks ratio of homologous *BraSWEET* genes was much lower than 1, indicating that these were strongly purified during the evolution. The *SWEET* duplicated gene pairs were separated from each other from 5 to 24 million years ago (MYA), except for *BraSWEET5.1* and *BraSWEET5.2,* on the one hand, and *BraSWEET14.2* and *BraSWEET10*, which were separated from each other by 3.1 and 70.8 MYA, respectively. Ks/2r (r = 1.5 × 10 − 8) was used for calculation.

**Figure 6:**
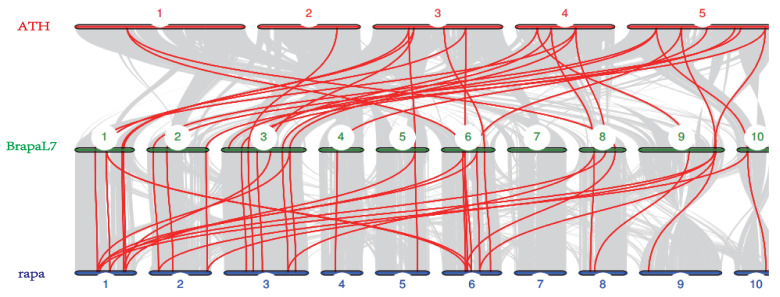
Synteny analysis of SWEET genes in *B. rapa* (L7), Arabidopsis, and Chinese cabbage. The gray lines in the background represent collinear blocks in genomes of *B. rapa* (BrapaL7), A. thaliana (ATH), and Chinese cabbage (rapa), and the red lines highlight collinear SWEET gene pairs.

Orthologous *BraSWEET* genes in the *B. rapa* genome were explored with those in the Chinese cabbage and *A. thaliana* genomes to clarify divergence in the evolution of *B. rapa*. In this study, collinearity was observed among SWEET family genes in *B. rapa* (L7), with 27 genes in *A. thaliana* and 37 in Chinese cabbage (Figure 6). According to these results, *B. rapa* has a close relationship with *A. thaliana* and Chinese cabbage; in particular, *SWEET* genes of Chinese cabbage have similar functions.

### 2.4. Analysis of Secondary and Tertiary Structures of BraSWEET Proteins

**Figure 7:**
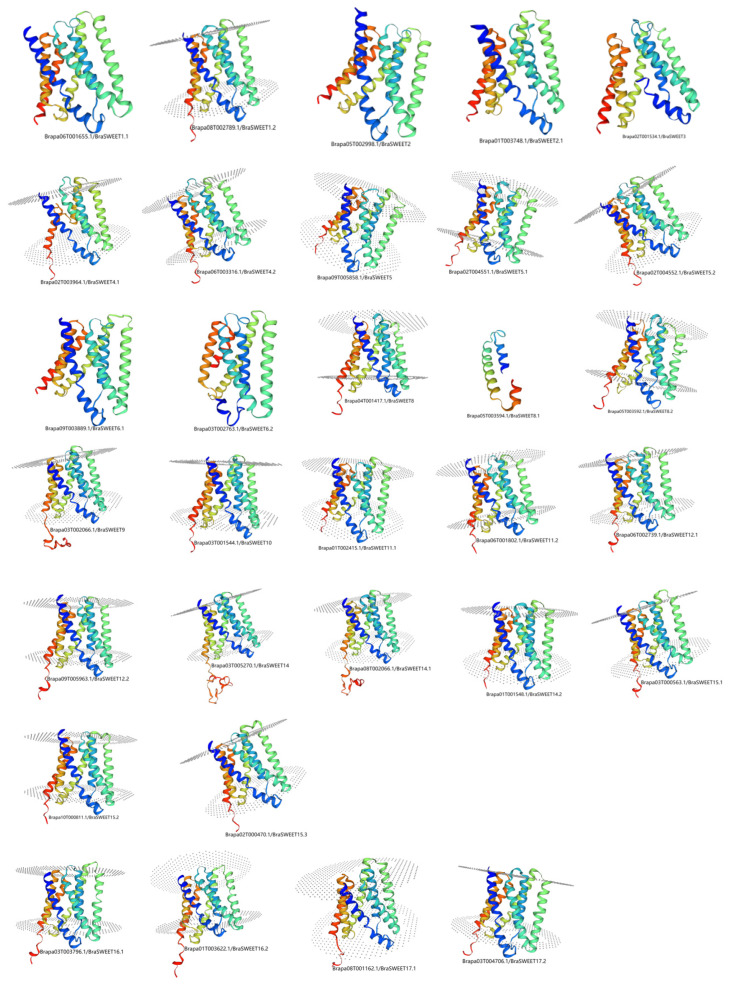
Predicted tertiary structure of BraSWEET proteins.

Varied percentages of alpha-helix (29.03–48.92%), extended chain (14.83–38.71%), β-turn (1.34–9.68%), and random coils (22.58–42.56%) were found in BraSWEET proteins. Nonetheless, in the same evolutionary clades, the secondary structures showed certain clustering, except for BraSWEET8.1/Brapa05T003594.1 (Table S1). Similar tertiary structures of 31 BraSWEET proteins were formed by folding of the secondary structures (Figure 7). There were structural differences in Group 2, including BraSWEET4.1/Brapa02T003964.1, BraSWEET6.2/Brapa03T002763.1, and BraSWEET8.1/Brapa05T003594.1.

### 2.5. Cis-Element Analysis for the BraSWEET Gene Family

**Figure 8:**
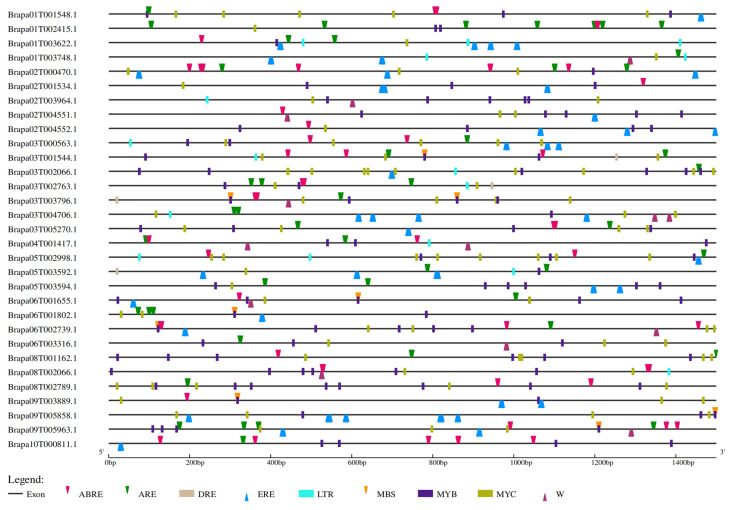
Cis-acting elements in the promoter regions of *BraSWEETs*. Cis-acting elements were identified by PlantCARE using upstream 1500 bp sequences of the *BraSWEETs*. Red inverted triangle, green inverted triangle, brown square, blue triangle, light blue square, orange inverted triangle, purple square, dark green square, dark red triangle, and red inverted triangle represent *ABRE*, *ARE*, *DRE*, *ERE*, *LTR*, *MBS*, *MYB*, *MYC*, and *W-Box*, respectively. The scale bar on the bottom indicates the length of promoter sequences.

Metabolic networks and regulatory mechanisms of *BraSWEET* genes were performed to understand the genetic functions using *cis*-acting elements in the 1.5 kb promoter sequences. There were four classes of cis-acting elements associated with growth and development, light response, phytohormone response, and stress response in the promoter region (Table S2). By focusing on the cis-acting elements related to stress response (Figure 8), all 31 gene promoter regions contained *MYC*, and 30 gene promoter regions included *MYB*, except for *BraSWEET2.1/Brapa01T003748.1*. Abscisic acid responsive element (*ABRE)* was found in 28 *BraSWEET* genes in multiple copies, except for *BraSWEET5/Brapa09T005858.1*, *BraSWEET8.1/Brapa05T003594.1*, and *BraSWEET17.2/Brapa03T004706.1*. All three types of cis-acting elements were associated with *ABA*-induced responses and are present on various resistance gene promoters. Ethylene-responsive element (ERE) was present in 24 *BraSWEET* genes. *LTR* (cis-acting element involved in low-temperature responsiveness) was found in 15 *BraSWEET* genes. Four gene promoters (namely *BraSWEET6.2/Brapa03T002763.1*, *BraSWEET8.2/Brapa05T003592.1*, *BraSWEET10/Brapa03T001544.1*, and *BraSWEET16.1/Brapa03T003796.1*) contained dehydration responsive element (*DRE)*, which were expressed under drought, high salt, and low-temperature stress [20,21]. Fifteen genes contained the *W-box* (WRKY transcription factor binding site) element, generally regulated by salicylic acid and presented in disease-resistance genes [22,23]. Twenty-nine genes harbored the antioxidant response element (ARE) and were involved in hypoxic conditions.

### 2.6. Protein–Protein Interaction Network for BraSWEETs

**Figure 9:**
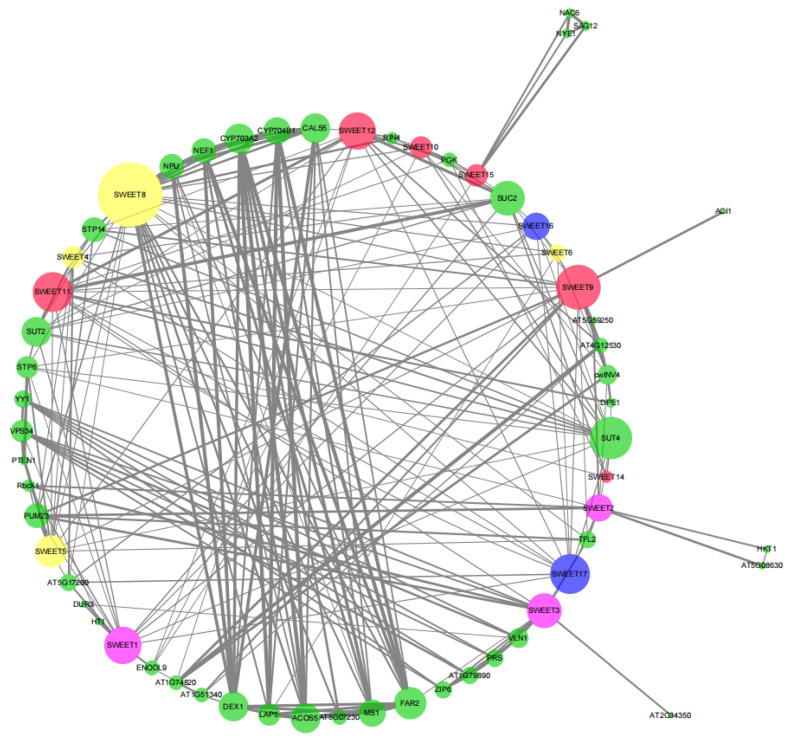
Predicted protein–protein interaction network for BraSWEET proteins. The network nodes represent proteins. The line width indicates the reliability of the interaction. The node size represents the number of proteins that interact with each other.

A BraSWEET interaction network was constructed for Arabidopsis orthologous proteins to explore the potential regulatory network and function of BraSWEETs. According to the protein–protein interactor predictions, some BraSWEETs could interact with other BraSWEETs; for instance, BraSWEET10 interacted with BraSWEET8 (Figure S2). BraSWEETs interacted with other proteins, suggestive of a common role (Figure 9). As indicated by these results, BraSWEETs are involved in regulating growth, development, and mediating biotic stress in plants.

### 2.7. Analysis of Transcriptional Expression of BraSWEET Genes During Overwintering

**Figure 10:**
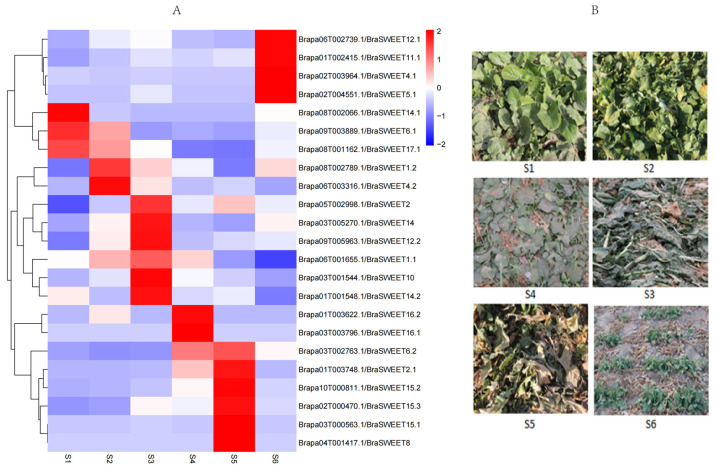
Expression profiles of 23 *BraSWEWTs* genes in different overwintering periods. (**A**) Heat map of *BraSWEWTs* genes in six periods of overwintering (S1–S6). (**B**) Plant growth map in different wintering periods (S1–S6).

The root collar tissues of L7 were selected to evaluate the expression levels of *BraSWEETs* in different overwintering stages. Twenty-three genes exhibited notable changes in expression (Figure 10). *Brapa08T002066.1/BraSWEET14.1*, *Brapa08T001162.1/BraSWEET17.1*, and *Brapa09T003889.1/BraSWEET6.1* were highly expressed in the S1 stage, and those of *Brapa08T001162.1/BraSWEET17.1* and *Brapa09T003889.1/BraSWEET6.1* lasted till the S2 stage. *Brapa08T002789.1/BraSWEET1.2* and *Brapa06T003316.1/BraSWEET4.2* were highly expressed in the S2 stage and decreased gradually. The expressions of *Brapa03T001544.1/BraSWEET10*, *Brapa09T005963.1/BraSWEET12.2, Brapa03T005270.1/BraSWEET14, Brapa01T001548.1/BraSWEET14.2,* and *Brapa05T002998.1/BraSWEET2* were high in the S3 stage. The expressions of *Brapa03T003796.1/BraSWEET16.1* and *Brapa01T003622.1/BraSWEET16.2* were the highest in the S4 stage. The expressions of *Brapa01T003748.1/BraSWEET2.1* and *Brapa03T002763.1/BraSWEET6.2* increased in the S4 stage. The expressions of *Brapa03T000563.1/BraSWEET15.1, Brapa10T000811.1/BraSWEET15.2, Brapa02T000470.1/BraSWEET15.3*, and *Brapa04T001417.1/BraSWEET8* were the highest in the S5 stage. *Brapa01T002415.1/BraSWEET11.1, Brapa06T002739.1/BraSWEET12.1, Brapa02T003964.1/BraSWEET4.1*, and *Brapa02T004551.1/BraSWEET5.1* were highly expressed in the S6 stage when the plant turned green. On the whole, when the temperature was above zero (4 °C), three *BraSWEET* genes (*Brapa08T002066.1/BraSWEET14.1, Brapa08T001162.1/BraSWEET17.1*, and *Brapa09T003889.1/BraSWEET6.1*) were expressed in response. Sixteen genes were highly expressed in sub-zero low-temperature stages (S2–S5 stages). When the temperature increased gradually (with the lowest air temperature of −1 °C) and the plants turned green, four genes, including *Brapa01T002415.1/BraSWEET11.1*, *Brapa06T002739.1/BraSWEET12.1*, *Brapa02T003964.1/BraSWEET4.1*, and *Brapa02T004551.1/BraSWEET5.1*, were expressed.

### 2.8. Subcellular Localization of BraSWEET10 Protein in Tobacco

**Figure 11:**
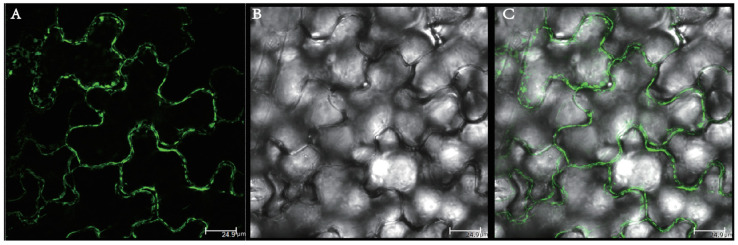
Subcellular localization of BraSWEET10 in tobacco. Treatment: 20% sucrose, 5–10 min. (**A**) Fluorescence image for BraSWEET10-GFP. (**B**) Bright field. (**C**) Merger of the first two images.

A previously constructed subcellular localization vector, 35S-BraSWEET10-GFP, was transiently expressed in tobacco leaves, and after 2–3 days of dark culture, the leaves were observed under a confocal microscope. Green fluorescence was localized in the cell membranes of the cells of the tobacco leaves (Figure 11), and we inferred that BraSWEET10 was localized to the cell membranes, consistent with the prediction. This result was further verified by other methods in the later stages.

### 2.9. Screening and Characterization of BraSWEET10 Overexpressing Transgenic A. thaliana 

**Figure 12:**
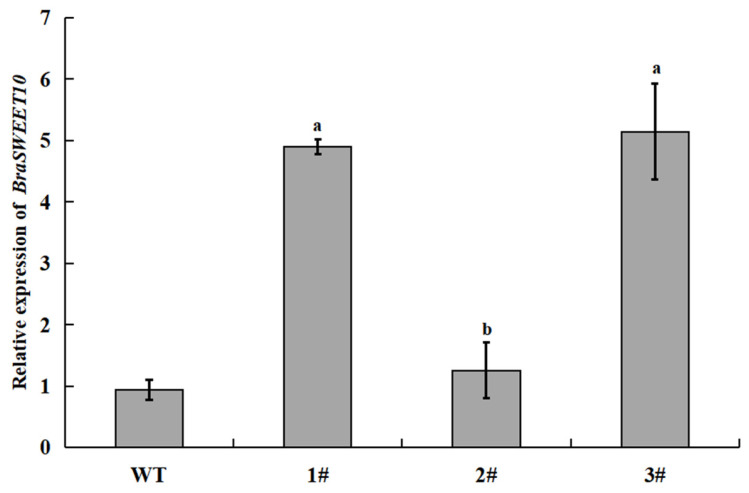
Expression level of *BraSWEET10* in *transgenic A. thaliana.* WT: wild type, 1#/2#/3#: *BraSWEET10* transgenic *A. thaliana*. ^a^ *p* < 0.01 vs. WT group, ^b^ *p* < 0.05 vs. WT group.

*A. thaliana* pure lines overexpressing the *BraSWEET10* gene were successfully obtained by the flower dipping method. *BraSWEET10* gene expression was characterized by qRT-PCR. As shown in Figure 12, among the *A. thaliana* pure lines obtained by screening, the relative expression of the *BraSWEET10* gene was 5-fold higher than that of the wild-type *A. thaliana.* plants No. 1 and No. 3; the expression in No. 2 plants was basically the same as that of the *A. thaliana* wild-type plants. Therefore, in the subsequent experiments, we selected No. 1 and No. 3 as experimental objects.

### 2.10. Resistance Analysis of BraSWEET10 Overexpressing A. thaliana Plants

**Figure 13:**
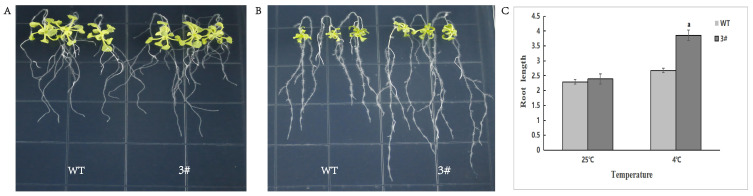
Root length of transgenic *A. thaliana* after low-temperature stress. WT: wild type, 3#: *BraSWEET10* transgenic *A. thaliana.* (**A**) Normal condition culture, (**B**) low-temperature (4 °C) treatment, (**C**) root length of *A. thaliana* plants after low-temperature treatment. ^a^ *p* < 0.01, 3# group vs. WT group.

Wild-type *A. thaliana* and transgenic plants were planted on MS plates and incubated vertically for 10 days at 4 °C for 12 h and normally for 7 days. Root lengths were measured, as shown in Figure 13. The root lengths of overexpressing *A. thaliana* plants without low-temperature treatment were the same as those of the wild type, and those of overexpressing *A. thaliana* plants were significantly longer than those of wild-type plants after low-temperature treatment. This indicated BraSWEET10’s potential involvement in the regulation of root development at low temperature.

## 3. Discussion

The first member of the SWEET gene family, named MtN3, was identified in the legume *Medicago truncatula* [24]. A new class of sugar transporters, called SWEETs, was discovered in 2010 by employing optical glucose sensors and are primarily involved in sugar efflux [6]. The functions of *SWEET* genes have been explored and studied in different plant species such as *A. thaliana,* wheat, cucumber, *alfalfa*, Chinese cabbage, sorghum, cassava, soybean, cotton, and sugarcane [25]. These studies have mainly focused on their role in growth and development, pollen development, and response to abiotic and biotic stress in plants [17,26,27,28,29,30,31,32,33,34,35,36,37]. L7 shows remarkably strong cold resistance, withstanding extremely low temperature, even sub-zero 32 °C. In this study, 31 *BraSWEET* genes were successfully identified from the *B. rapa* whole-genome data. These genes were classified into Groups 1–4, conforming to the previous clades of *A. thaliana* and maize. According to expression profile analysis, 23 *BraSWEET* genes were differentially expressed during overwintering, and these might be involved in coping with overwintering.

### 3.1. Characteristics of the BraSWEET Gene Family

Most predicted SWEET proteins possess seven transmembrane helices, forming two MtN3 domains [6,7,38,39]. As shown in this study, most BraSWEET proteins contained seven transmembrane helices, and TM1-3 and TM5-7 constituted the two MtN3 domains. This domain was highly evolutionarily conserved. Brapa05T003594.1/BraSWEET8.1 was a semi-SWEET, containing only one MtN3 domain. Thirty-one members of the BraSWEET family are localized to the plasma membrane [6].

BraSWEETs can be divided into four clades, and Groups 2 and 3 were the main clades in most plants. This feature was confirmed in this study. Genes 10 and 11 were defined in Groups 2 and 3, respectively, indicating their vital function. As indicated in previous studies on the characteristics of sugar transport, the SWEET proteins in Groups 1, 2, and 4 transported hexoses, including glucose, fructose, and galactose, while Group 3 encoded proteins controlling the efflux of sucrose [40,41,42]. The structure of *BraSWEET* genes was closely related to their function, mainly in the distribution of conserved motif (Figure 2C). Motif2-3-5-1-4-4 was found in Groups 1, 2, and 4, and Motif2-3-6-5-1-4-7 was found in Group 3, indicating the diversity and functional differentiation of *BraSWEET* genes through evolution. Furthermore, the number and location of intron–exon were correlated with the function. Most *BraSWEET* genes had six exons and five introns, as in other plants, including soybean [43], cucumber [44], and *B. rapa* [16,35]. The clustered *BraSWEET* genes exhibited roughly the same gene structure. For instance, *BraSWEET15.1*, 15.2, and 15.3 in Group 3 had a special structure comprising one long intron and four short introns; *BraSWEET17.1*/*Brapa08T001162.1* and *BraSWEET17.2/Brapa03T004706.1* in Group 4 possessed a special structure with three short introns and two long introns (Figure 2B).

### 3.2. Evolutionary Analysis of the BraSWEET Gene Family

Whole-genome triplication (WGT) has been carried out since the separation of *Brassica* from *A. thaliana* was discovered in 2016 [45]. A total of 31 *BraSWEET* genes were found in *B. rapa* (L7), which is 1.82 times higher than the 17 *AtSWEET* genes (Table 1). *BraSWEET* genes were predicted to expand by triplication. Five groups of *BraSWEET* lost genes during evolution; for example, *AtSWETT7* and *AtSWETT13* have no homologs in the *B. rapa* genome, while *AtSWETT3*, *AtSWETT9*, and *AtSWETT10* have only one homolog each (Figure 1). Due to the increase in *BraSWEET* genes, the SWEET gene family exhibits more functions. Simultaneously, there may be functional redundancy or differentiation among its members. According to this study, winter *B. rapa* expanded by tandem duplication and fragment duplication, and a total of 18 pairs of fragment-repetitive genes have been identified. Furthermore, 27 and 37 *BraSWEET* genes indicated collinearity with the *A. thaliana* and *B. rapa* genome, respectively. This suggests similar functions of the *SWEET* genes. *BraSWEET* genes were found to be under purifying selection, consistent with previous evolutionary analyses [8,16,43,46].

### 3.3. Prediction of Gene Function Based on Protein Interaction and Promoter Analysis

SWEET8 interacts with several pollen development-related proteins, such as DEX1, NUP, CYP703A2, CYP704B1, and ACOS5 [24,25,26]. BraSWEET2, 3, 8, and 9 interact with translation regulation-related protein, PUM23 [27]. BraSWEET4, 10, 11, and 12 can interact with sucrose transporters SUT2 and SUT4. AtSUT4 regulates sucrose balance and sink via the *ABA* pathway, which is closely associated with low-temperature, high-salt, and osmotic stress in *A. thaliana* [28]. BraSWEET15 can interact with the plant senescence regulatory-related protein SAG12 [29] and with the plant stress resistance-related transcription factor *NAC*6 [30]. BraSWEET10 might interact with the pathogen response-related protein RIN4 and with the programmed death-related protein PGK [31,32,33]. Cis-acting elements of *BraSWEET* promoter regions were mainly divided into four classes, namely growth and development regulation, light response, hormone response, and stress response elements (Figure 8, Table S2). Among stress response elements, three *ABA*-induced response elements were detected, namely the *MYB* (CCAATbox) element, *MYC* (CACATGbox) element, and *ABRE* homeopathic element. Other stress response elements were identified, including *W-box* involved in salicylic acid and disease stress response, *ERE* involved in ethylene response, *LTR*, *DRE*, and *ARE*. In this study, BraSWEET proteins were found to interact with pollen development proteins, senescence regulation-related proteins, stress resistance-related transcription factors, pathogen response-related proteins, and programmed death proteins. According to these results, BraSWEETs may be involved in regulating the growth, development, and biotic stress adaption of plants through hormonal regulation.

### 3.4. Analysis of BraSWEET Expression During Overwintering

Plants can transfer more nutrients from the aboveground organ to the underground survival organ (root collar tissues) during overwintering. After regreening of the aboveground part, some low-temperature stress genes function at low temperature. From the transcriptome data, differential expression of *BraSWEET* genes during overwintering was detected [14]. *SWEET6*, 14, and 17 were highly expressed in the pre-winter stage (S1), and 16 *BraSWEET* genes (*BraSWEET1.1*, *BraSWEET1.2*, *BraSWEET2*, *BraSWEET2.1*, *BraSWEET4.2*, *BraSWEET6.2*, *BraSWEET8*, *BraSWEET10*, *BraSWEET12.2*, *BraSWEET14*, *BraSWEET14.2*, *BraSWEET15.1*, *BraSWEET15.2*, *BraSWEET15.3*, *BraSWEET16.1*, and *BraSWEET16.2*) were differentially expressed in the overwintering stages (S2-S5). According to a report, some of these genes in other species provide cold stress resistance. For example, overexpression of *AtSWEET16* and *AtSWEET17* enhances cold resistance in *A. thaliana* [1,47]. *AtSWEET15* expression is induced under high-salt and drought stress and upregulated under cold stress [10,48]. *GhSWEET2a* and *GhSWEET2b* are upregulated under cold treatment [35]. *CsSWEET16* can respond to cold stress and export fructose from vacuoles, and *CsSWEET2*, *CsSWEET3*, and *CsSWEET6* are associated with cold stress [49,50]. Moreover, *MtSWEET1a* is inhibited under cold and drought stress but induced under salt stress [36]. Mutants of *AtSWEET11* and *AtSWEET12* affected freezing tolerance in *A. thaliana* [13]. Nonetheless, some genes, such as *BraSWEET8*, *BraSWEET10*, and *BraSWEET14*, were first found to be associated with freezing resistance. As demonstrated in previous studies, *AtSWEET8* and *AtSWEET13* are crucial for pollen nutrition and are expressed in the tapetum and anthers, respectively. *AtSWEET9* serves as a sucrose transporter for nectar production [42]. In rice, *OsSWEET14* is crucial for reproductive organ development [51]. Whether these genes are important for the successful overwintering mechanism in winter *B. rapa* merits further investigation based on the flowering of plants. In the regreening stage (S6), the expressions of *BraSWEET4*, *BraSWEET5*, *BraSWEET11*, and *BraSWEET12* were upregulated, suggesting that the aboveground parts began to re-synthesize carbohydrates, further transferring them to the root collar tissues and ensuring their continuous resistance to low-temperature stress. During the overwintering of winter rapeseed, the aboveground tissues died, suggesting that no more sugars were synthesized. Several *BraSWEET* genes were differentially expressed in the root collar tissues (including the shoot apical meristem). These results indicate that some *BraSWEET* genes are playing a role in the concentration of sugars in the root collar tissues, thus better resisting low-temperature stress and inducing flowering.

In general, 31 *BraSWEET* genes were identified in the L7 genome. These genes acted as sugar transporters and played essential roles in the growth and development of plants and in mediating responses to abiotic stresses. In this study, the evolutionary relationships and overwintering expression patterns of the *BraSWEET* genes were explored. Moreover, we hypothesized that some *BraSWEET* genes were associated with frost resistance and some genes were involved in overwintering memory mechanisms. The results provide insights into the potential functions and characteristics of the *BraSWEET* genes and lay the foundation for future studies on the biological roles of the *BraSWEET* genes in winter *B. rapa*.

### 3.5. Functional Analysis of BraSWEET10

The promoter region of the *BraSWEET10* gene contains several cis-acting elements related to stress response, phytohormones, light response, circadian rhythms, and promoter. It is actively involved in hormone regulation and stress tolerance. The BraSWEET10 protein is related to sucrose transporter proteins, jasmonic acid (JA) biosynthesis-associated proteins, bud meristem regulation, programmed death proteins, sex proteins, programmed death proteins, and sex differentiation-related proteins, which may be involved in plant sugar transport, flower differentiation, and pollen development.

We overexpressed *BraSWEET10* in *A. thaliana* and observed their phenotypes under low temperature. The root length of *BraSWEET10*-overexpressing *A. thaliana* plants was significantly longer than WT plants under low-temperature treatment, suggesting that *BraSWEET10* may be involved in the regulation of root development at low temperature to improve its freezing tolerance by enhancing root development. However, its function related to the regulation of flowering has not been thoroughly investigated in winter *B. rapa*.

## 4. Materials and Methods

### 4.1. Identification of the SWEET Gene Family in B. rapa

The hidden Markov model (HMM) profiles of the SWEET domain (PF03083) were downloaded from the Pfam database (http://pfam.xfam.org/ (accessed on 21 March 2024)) and used to search the MtN3_saliva domains in the *B. rapa* (L7) proteome by employing the HMMER software (3.3.2) (http://hmmer.org/ (accessed on 22 March 2024)) [36,52]. With default parameters, a *p*-value of 0.01 was the significance threshold. ClustalW was used to compare all extracted candidate genes possibly containing MtN3_saliva domain, and the HMM model was reconstructed (with a value below 0.001). Redundant sequences were removed. By applying the SMART program (http://smart.embl-heidelberg.de// (accessed on 22 March 2024)), the core sequence of the MtN3_saliva domain was identified. After multiple comparisons, 31 *SWEET* genes were finally confirmed in the rapeseed genome. Afterward, by employing the tools in ExPasy (http://web.expasy.org/protparam// (accessed on 24 March 2024)), the sequence length, molecular weight, and isoelectric point predictions of the confirmed SWEET proteins were obtained [53].

### 4.2. Multiple Sequence Alignment and Phylogenetic Analysis of BraSWEET Genes

The full-length amino acid sequences of AtSWEETs (data from the TAIR website) and BraSWEETs were used for phylogenetic analysis. Multi-sequence alignment was performed by adopting the clustalW program, and an unrooted neighbor-joining phylogenetic tree was constructed using the MEGA7 (v7.0.14) software. All parameters used were default, and the bootstrap was 1000 [54,55]. Based on the topological structure of the phylogenetic tree and the classification of AtSWEETs, BraSWEETs were divided into four groups.

### 4.3. Analysis of Transmembrane Structure, Conserved Motif, and Gene Structure

Transmembrane helices of the BraSWEET family proteins were predicted by employing TMHMM2.0 (http://www.cbs.dtu.dk/services/TMHMM/ (accessed on 24 March 2024)) with their amino acid sequences [56]. Twenty-four members of the BraSWEET proteins had typical seven trans-membrane helices. The MEME software (v4.3.12) (https://meme-suite.org/ (accessed on 24 March 2024)) was adopted to predict the common conservative motifs of 31 BraSWEET proteins. The parameters were set as follows: optimal sequence width was set to 6 and 50; the maximum number of designed motifs was seven, and the iterative cycle was set as default [57]. The structure of *BraSWEET* genes was analyzed using GSDS (http://gsds.gao-lab.org/ (accessed on 25 March 2024)) [58].

### 4.4. Chromosomal Location and Gene Duplication of BraSWEET Genes

Based on the physical location of 31 *BraSWEET* genes on chromosomes, the MapChart software (v5.4.6) was used to map the genes [59]. Tandem duplications and segmental duplications of the BraSWEET gene family were searched to analyze the pattern of gene evolution. Using BLAST (v2.16.0), the region of tandem duplication was found. By employing MCScan X (default parameter), segmental duplications were examined [60]. The time of occurrence of segmental duplication events for homologous genes was calculated as T = Ks/2r, (r = 1.5 × 10^−8^) and expressed in MYA.

### 4.5. Analysis of the Secondary and Tertiary Structures of BraSWEET Proteins

The secondary structure of the BraSWEET proteins was predicted using the self-optimized prediction method with alignment (SOPMA) server (https://npsa-prabi.ibcp.fr/cgi-bin/npsa_automat.pl?page=/NPSA/npsa_sopma.html (accessed on 29 March 2024)), with a similarity threshold of 8. The SWISS-MODEL (https://swissmodel.expasy.org/) was adopted to identify the known structure with over 30% consistency with the BraSWEET sequences, and the known structure was deemed as a template to construct the target protein structure, followed by drawing the 3D structures of the 31 BraSWEET proteins. The structures are available at (https://saves.mbi.ucla.edu/).

### 4.6. Promoter Analysis for BraSWEETs

Generally, the 1500 bp sequence upstream of the gene was extracted using in-house Perl scripts and considered the promoter region, used for cis-acting element prediction of the BraSWEET family. Using the Plant CARE website (http://bioinformatics.psb.ugent.be/webtools/plantcare/html/ (accessed on 3 April 2024)), several cis-acting elements were obtained by analyzing FASTA sequences. By rigorous screening, some cis-acting elements possibly involved in hormonal processes, growth regulation, and stress response were retained. The GSDS (http://gsds.cbi.pku.edu.cn/) was applied to analyze the cis-regulatory element of the BraSWEET family [58].

### 4.7. Plant Materials, Field Trails, Sample Collection, and Transcriptional Expression Analysis of BraSWEET Genes During Overwintering by RNA-Seq Data Analysis

L7 was selected as the plant material and planted in the Gansu Research Center of Rapeseed Engineering and Technology located in Shangchuan town, Yongdeng county, Lanzhou City, Gansu province (longitude: 103.67° E; latitude: 36.05° N; altitude: 2180 m). From autumn 2019 to spring 2020, samples were collected at six time points, namely 9 October 2019 (L7_S1), 2 November 2019 (L7_S2), 24 November 2019 (L7_S3), 15 December 2019 (L7_S4), 4 January 2020 (L7_S5), and 25 April 2020 (L7_S6). The first sampling day was 9 October 2019, and the lowest air temperature was 4 °C. From 2 November 2019, to 4 January 2020, the average lowest air temperature was −13 °C. On the last sampling day of 25 April 2020, the lowest air temperature was −1 °C, and the winter *B. rapa* regreened. As roots are critical tissues and the only living organs in winter *B. rapa* required for survival during overwintering, the root collar tissues (a 5 mm section below the cotyledon nodes), including the shoot apical meristem (a 3 mm section above the crown base), were selected in the six sampling stages with the same proportion for *RNA-Seq* analyses. Each material was collected from more than three plants. For the specific method, please refer to Lijun Liu’s treatment protocol [14]. *RNA-Seq* data were processed with TBtools (v0.655) and plotted as a heatmap to visualize the changes in *SWEET* gene expression (https://github.com/CJ-Chen/TBtools (accessed on 12 April 2024)).

### 4.8. Construction of the Overexpression Vector

Specific primers (*BraSWEET10-F1/R1*) flanking the *BraSWEET10* gene were designed using Primer Premier 5.0, with XbaI and KpnI restriction sites engineered at their 5′ ends. The primer sequences used are as follows:

*BraSWEET10-F1*: CGGGGGACGAGCTCGGTACCATGGCGGTTTCAATAGTCG.

*BraSWEET10-R1*: ACCATGGTGTCGACTCTAGATTCTTGGATATAAGAAGCAT.

The PCR-amplified *BraSWEET10* fragment was ligated into the pCAMBIA2300-35S vector via XbaI/KpnI digestion. Positive clones were verified by sequencing and transformed into *Agrobacterium tumefaciens* GV3101 for subsequent use.

### 4.9. Subcellular Localization of the BraSWEET10 Protein in Tobacco (Nicotiana benthamiana)

The pCAMBIA2300-BraSWEET10-GFP-positive *Agrobacterium* spp. was transiently transformed into tobacco, and the infested tobacco was placed in 23 °C in the dark for 2–3 days. Tobacco leaves were cut and placed under a laser confocal microscope (LSCM 800), and the fluorescence localization of GFP was observed at an excitation wavelength of 488 nm.

### 4.10. Transformation of A. thaliana Using Overexpression Vectors

Wild-type *A. thaliana* was used as a background for the flower-dipping method and screening pure combinations. The specific method was as follows:

The constructed *Agrobacterium* spp. containing the *BraSWEET10* gene was harvested when the OD600 of the activated spp. reached 0.8. The sample was centrifuged at 4000–6000 rpm for 15–10 min at room temperature; the supernatant was discarded, and 50 mL of infiltration medium (1/2 MS, 5% sucrose, 0.02% surfactant, 0.1% MES, pH of about 5.7) was used to suspend the pellet. This step was repeated twice and poured into a clean petri dish. Pod-stage *A. thaliana* seedlings without fruit pods were selected for infestation, and they were poured into the infestation medium for 30–60 s. After removal, they were incubated for 24 h away from light and then incubated normally until T0 generation seeds were collected.

The T0 generation seeds were planted on the MS solid medium containing Kan, and the Kan-resistant transgenic *A. thaliana* with green cotyledon color was transplanted and cultured to collect the T1 generation seeds. The T1 generation seeds were planted on an MS solid medium containing Kan and cultured. The T2 generation was obtained by collecting the seeds from a single plant. The T2 generation seeds were tested on the MS medium containing Kan again for segregation ratio. Fully viable seedlings and their corresponding T2 generation seeds were considered pure, and the transgenic pure strain was obtained. Total RNA was extracted from the leaves of the transgenic plants, and the cDNA was reverse transcribed to identify the overexpression of the target gene by qRT-PCR.

### 4.11. Phenotypic Observations of Transgenic A. thaliana

Wild-type and transgenic *A. thaliana,* approximately 10 d after germination were incubated at 4 °C in an incubator for 12 h, and the culture was continued for 7 d under normal conditions to observe the root length of the plants.

## Figures and Tables

**Table 2:** Identification of substitution rates for homologous *BraSWEET* genes.

Orthologous Gene Pairs	Non-Synonymous Substitution Rate (Ka)	Synonymous Substitution Rate (Ks)	Ka/Ks	Duplication Date (MYA)
*Brapa06T001655/BraSWEET1.1*	0.0302	0.2264	0.1332	7.55
*Brapa08T002789/BraSWEET1.2*				
*Brapa01T003748/BraSWEET2.1*	0.0714	0.3188	0.2241	10.63
*Brapa05T002998/BraSWEET2*				
*Brapa02T003964/BraSWEET4.1*	0.0375	0.2520	0.1488	8.40
*Brapa06T003316/BraSWEET4.2*				
*Brapa02T004551/BraSWEET5.1*	0.0764	0.0928	0.8239	3.09
*Brapa02T004552/BraSWEET5.2*				
*Brapa02T004551/BraSWEET5.1*	0.1144	0.2685	0.4261	8.95
*Brapa09T005858/BraSWEET5*				
*Brapa03T002763/BraSWEET6.2*	0.2182	0.5115	0.4267	17.05
*Brapa09T003889/BraSWEET6.1*				
*Brapa01T002415/BraSWEET11.1*	0.0738	0.2497	0.2955	8.32
*Brapa06T001802/BraSWEET11.2*				
*Brapa06T001802/BraSWEET11.2*	0.0777	0.7063	0.1100	23.54
*Brapa06T002739/BraSWEET12.1*				
*Brapa06T002739/BraSWEET12.1*	0.0460	0.1753	0.2625	5.84
*Brapa09T005963/BraSWEET12.2*				
*Brapa03T005270/BraSWEET14*	0.0431	0.2317	0.1861	7.72
*Brapa08T002066/BraSWEET14.1*				
*Brapa01T001548/BraSWEET14.2*	0.0340	0.2175	0.1565	7.25
*Brapa03T005270/BraSWEET14*				
*Brapa01T001548/BraSWEET14.2*	0.0323	0.2122	0.1521	7.07
*Brapa08T002066/BraSWEET14.1*				
*Brapa01T001548/BraSWEET14.2*	0.4723	2.1231	0.2224	70.77
*Brapa03T001544/BraSWEET10*				
*Brapa03T000563/BraSWEET15.1*	0.0782	0.2395	0.3263	7.98
*Brapa10T000811/BraSWEET15.2*				
*Brapa01T003622/BraSWEET16.2*	0.0452	0.3249	0.1392	10.83
*Brapa03T003796/BraSWEET16.1*				
*Brapa01T003622/BraSWEET16.2*	0.1716	0.6453	0.2659	21.51
*Brapa03T004706/BraSWEET17.2*				
*Brapa02T000470/BraSWEET17.2*	0.0967	0.2848	0.3394	9.49
*Brapa03T000563/BraSWEET15.1*				
*Brapa02T000470/BraSWEET17.2*	0.0937	0.2711	0.3458	9.04
*Brapa10T000811/BraSWEET15.2*				
*Brapa03T004706/BraSWEET17.2*	0.0520	0.1836	0.2831	6.12
*Brapa08T001162/BraSWEET17.1*				

## Data Availability

Data are contained within the article and Supplementary Materials.

## Supplementary Materials

The following supporting information can be downloaded at: https://www.mdpi.com/article/10.3390/ijms26062398/s1.
