# Supplementary material for: Genome-Wide Identification of the SWEET Gene Family and Functional Analysis of BraSWEET10 in Winter B. rapa (Brassica rapa L.) Under Low-Temperature Stress
Source: Int J Mol Sci. 2025 Mar 7;26(6):2398. doi: 10.3390/ijms26062398 (PMC11942336; doi:10.3390/ijms26062398)
Supplement: Supplementary file 1 [file ijms-26-02398-s001.zip › supplementary materialsú¿figureS1,S2ú⌐.pdf]

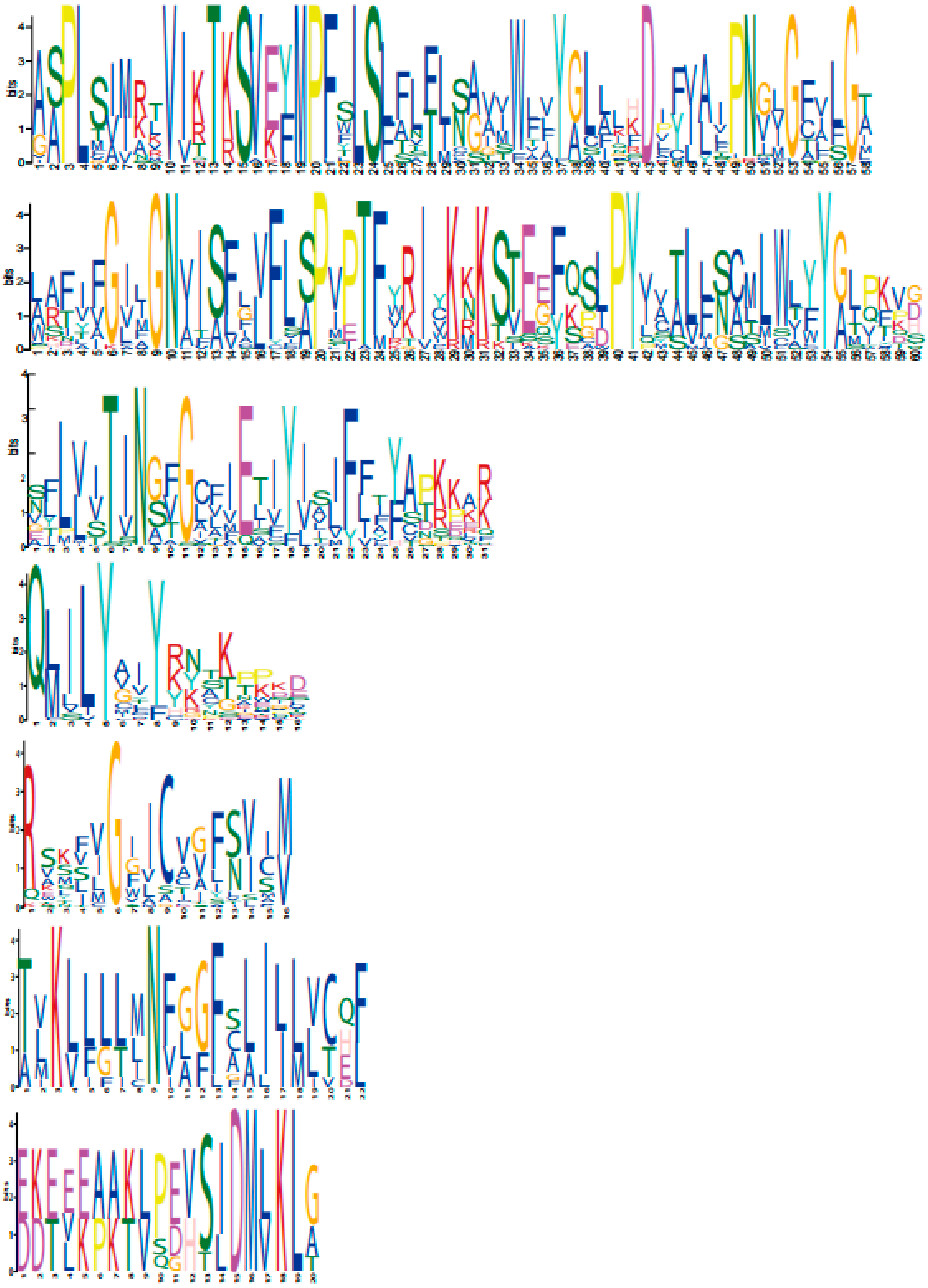

Figure S1. Sequence logos about the seven motifs of the BraSWEET proteins in *Brassica rapa* L. Motifs are displayed by stacks of letters at each site. The x-axis represents the width of motif and y-axis represents the bits of each letter, respectively. The height of each letter in a stack is the probability of the letter at that site.

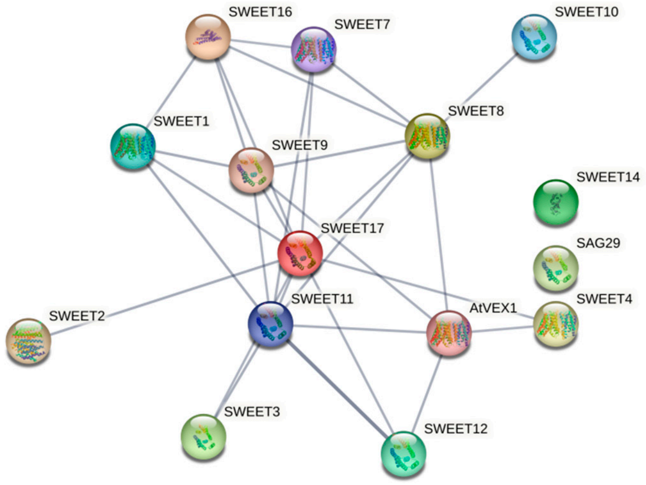

Figure S2. Protein interaction network of BraSWEET in *Brassica rapa* L.
